# Epidemiology of influenza from 2017 to 2022 in a national children’s regional medical center

**DOI:** 10.1186/s12887-025-05416-y

**Published:** 2025-03-27

**Authors:** Jiani Shan, Xin Yang, Tianlin Wang

**Affiliations:** 1 Outpatient Department, The Children’s Hospital, Zhejiang University School of Medicine, Hangzhou, 310052 China; 2 Department of genetic metabolism, The Children’s Hospital, Zhejiang University School of Medicine, Hangzhou, 310052 China; 3 The Children’s Hospital, Zhejiang University School of Medicine, Hangzhou, 310052 China

**Keywords:** Epidemiology, Children, Influenza, China

## Abstract

**Background:**

To examine the changes in influenza types (A/B), including influenza A subtypes (A(H1N1) / A(H3N2)) and influenza B lineages (B/Victoria and B/Yamagata) in children in Hangzhou City, China.

**Methods:**

This retrospective study was conducted in a national children’s regional medical center, using data from children screened for influenza between January 2017 and December 2022.

**Results:**

6775 patients (3 months to 14 years, 54.16% boys) were identified to have influenza-like symptoms. Among all patients, 905 (13.36%) patients were positive for the influenza virus. The number of patients positive for influenza was 222 (21.31%), 129 (12.40%), 270 (25.71%), 59 (5.15%), 37 (3.03%), and 188 (14.72%) from 2017 to 2022, respectively. The prevalence of influenza was higher in the more than 6 years old (*n* = 332, 23.23%) and 3–6 years old (*n* = 397, 13.18%) than in the under 3 years old (*n* = 176, 7.54%) groups, indicating that the influenza infection might increase with age. A/H3N2 infection was highest in the under 3 years old and 3–6 years old age groups while B/Victoria infection was highest in the more than 6 years old age group. The positive rates of influenza A (7.41% vs. 7.22%, *P* = 0.767) and B (5.47% vs. 5.94%, *P* = 0.407) among boys and girls did not have much difference The seasonal characteristics showed that, among patients with influenza-like symptoms, winter and spring were the dominant flu season in Hangzhou. The most common virus subtypes were B/Victoria in the spring and A/H3N2 in other seasons. The influenza positive rates among different seasons were different, in winter it was higher compared with the other seasons except for 2021 and 2022, results also revealed that influenza A/H3N2 had a relatively high prevalence in summer.

**Conclusions:**

The influenza viruses infection rate in 3 months to 14 years participants ranged from 3.03 to 25.7%, and the positive rate increased with age. No significant difference was observed in different sexes and subtypes of influenza. However, the relatively high prevalence of influenza A/H3N2 and high prevalence of all four subtypes indicate more attention to influenza infection should be paid in summer and winter.

**Supplementary Information:**

The online version contains supplementary material available at 10.1186/s12887-025-05416-y.

## Background

Influenza in children is a major cause of morbidity and mortality worldwide. One study showed that influenza was the most common (8.27%) respiratory virus among acute respiratory infection child patients in China [1], while in the USA, the predicted incidence of symptomatic influenza varies from 3–11% [2], and the annual epidemics in adults and children are associated with estimated 3–5 million cases of severe illness and about 290,000-650,000 deaths [3–5], and these facts also lead to the high economic burden of influenza [6, 7]. Also, influenza infection prevalence showed a seasonal change pattern in temperate countries, with peaks during the winter months, but it has sustained activity throughout the year in tropical climates [4, 5].

Influenza is characterized by its short incubation period, rapid spread, and high infectivity [8], especially in the child population [9]. Influenza surveillance data showed that 95.2% of influenza outbreaks occurred in mainland China’s primary and secondary schools and daycare centers in 2016–2017 [10]. The symptoms include sudden high fever, sore throat, muscle pains, headache, and cough [5, 11]. According to previous studies, children aged 6–59 months, children who have chronic pulmonary, cardiovascular, renal, hepatic, neurologic, hematologic, or metabolic disorders, immunosuppression status, children and adolescents who are receiving long-term aspirin therapy, adolescents who are or will be pregnant during the influenza season, residents of long-term care facilities, and children with morbid obesity are all with increased risk for influenza [3, 12–15]. In child patients with other systematic diseases, the symptoms of influenza could be even higher, Quertermous showed that influenza associated neuropsychiatric complications had a higher incidence in children with underlying neurologic or psychiatric conditions [16]; while Gomersall et al. showed that children exposed to polycyclic aromatic hydrocarbons are at a higher risk of developing respiratory diseases (including influenza) [17]. All these results exhibited that influenza still possessed a great threat to the child population.

Influenza viruses are divided into three types (A, B, and C) based on genetic and antigenic differences, type A can be further classified into subtypes based on the differences in hemagglutinin (H) and neuraminidase (N) located at capsid surface [18], and the two major types are A/H1N1 and A/H3N2 [19, 20], type B are classified into B/Victoria and B/Yamagata [21] and type C has no clinical significance in human populations [22].

Influenza viruses infections are a continuous and severe global threat to humans. Antigenic drift is the underlying mechanism involved in the evolution of the virus and is responsible for new strains of influenza appearing each year, while antigenic shift is an abrupt, major change resulting in a new subtype that may cause the pandemic, both mechanisms could make previous immunization (either through vaccination or a previous influenza infection) partially or completely obsolete against the new strain [4, 23]. Therefore, epidemiological surveillance is paramount for influenza management. The goal of influenza surveillance is to provide timely and high-quality data. These data allow for monitoring influenza activity levels and trends, tracking influenza virus variations, detecting new influenza viruses, making early warnings, and recommending global and national influenza vaccine strains.

Therefore, this study aimed to examine the changes in influenza strain from January 2017 to December 2022 in pediatric patients in Hangzhou City, China.

## Methods

### Study design and patients

This retrospective study was conducted at a national children’s regional medical center, the Children’s Hospital of Zhejiang University School of Medicine, an influenza surveillance sentinel hospital, using data from children screened for influenza between January 2017 and December 2022. The inclusion criteria were: (1) Patients admitted at the fever clinic, with influenza-like symptoms, including fever (≥ 38 °C) and acute respiratory symptoms such as cough or sore throat; (2) aged 0–18 years.

This work has been carried out in accordance with the Declaration of Helsinki (2000) of the World Medical Association. The study protocol and standardized data collection form were approved by the Ethics Committee of The Children’s Hospital of Zhejiang University School of Medicine. This study collected the medical records of the corresponding patients and carried out a retrospective study, which has minimal risk to the patients. The privacy of the patients was protected, and the interests of the patients were not infringed. All data were anonymized once extracted from the charts and databases. Therefore, the requirement for individual consent was waived by the ethics committee of The Children’s Hospital of Zhejiang University School of Medicine.

### Data collection and definition

All data were collected from the weekly disease report (20 cases per week according to the local policy) on influenza surveillance during the study period. The routine procedure at the fever clinic during the study period was to collect throat swabs from patients with influenza-like symptoms. The specimens were stored at 4 °C and delivered to the Center for Disease Control in Hangzhou within 24 h after collection, where influenza virus nucleic acid detection and viral genotyping were performed using a real-time PCR assay kit (Suoshi Biotechnology, Co., Ltd, Jiangsu, China). The data of the influenza virus nucleic acid detection and results of real-time PCR were collected. The demographic characteristics [including age, gender, and admission time (in which seasons)] were also collected from the electronic medical record. The seasons in Hangzhou City are divided based on the weather: spring (March-May), summer (June-August; the hottest season), autumn (September-November), and winter (December-February; the coldest season). In the present study, we further classified participants according to their age information, participants < 3 years were defined as infants, 3–6 years were pre-school, and those > 6 years were defined as school age, by adopting this criterion, we could better understand the distribution of influenza in each group [24]. 

### Statistical analysis

The data were analyzed using SPSS 19.0 (IBM, Armonk, NY, USA). The relationship between the viral detection rate and different age groups, years, or seasons was determined by the trend chi-square test. The rates were compared using the chi-square test, and the Cochran-Armitage trend test was used to study the trends. Two-tailed *p*-values < 0.05 were considered statistically significant.

## Results

**Fig. 1:**
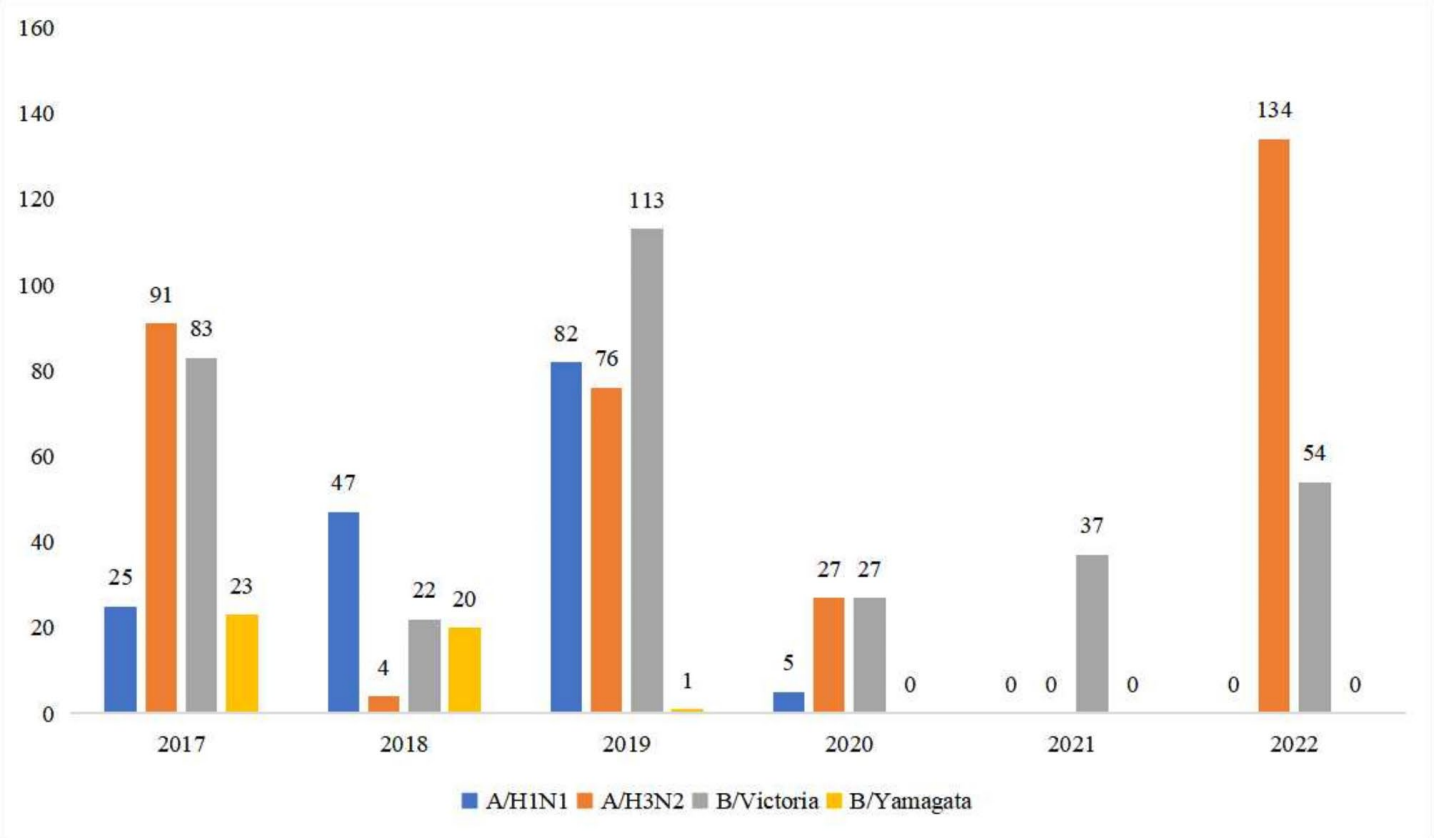
Types of influenza viruses circulating between 2017 and 2022

Between 2017 and 2022, 6775 patients were identified to have influenza-like symptoms. They were from 3 months to 14 years of age. There were 3618 (54.16%) boys. Among the 6775 patients, 905 (13.36%) patients were positive for the influenza virus. The number of patients positive for influenza from 2017 to 2022 was 222 (21.31%), 129 (12.40%), 270 (25.71%), 59 (5.15%), 37 (3.03%), and 188 (14.72%), respectively (Table 1), showing a statistically significant difference (*P* < 0.001). The distributions of the subtypes of influenza virus, including influenza virus A/H1N1 (A/H1N1), influenza virus A/H3N2, influenza virus B/Victoria, influenza virus B/Yamagata, between 2017 and 2022 are shown in Fig. 1. The A/H1N1 was only observed between 2017 and 2020, and the B/Yamagata was only present from 2017 to 2018. For A/H3N2 and B/Victoria, they were observed from 2017 to 2022 (except A/H3N2 in 2021), the oscillation amplitude of A/H3N2 was higher and the number of B/Victoria was more stable.

**Table 1:** Influenza surveillance data in children, 2017–2022

Year	Influenza-like symptoms, *n*	Positive cases, *n*	Positive rate*, %
**2017**	1042	222	21.31
**2018**	1040	129	12.40
**2019**	1050	270	25.71
**2020**	1146	59	5.15
**2021**	1220	37	3.03
**2022**	1277	188	14.72
**Total**	6775	905	13.36

*: The positive rate was calculated as the number of positive cases divided by the number of influenza-like symptom cases

**Fig. 2:**
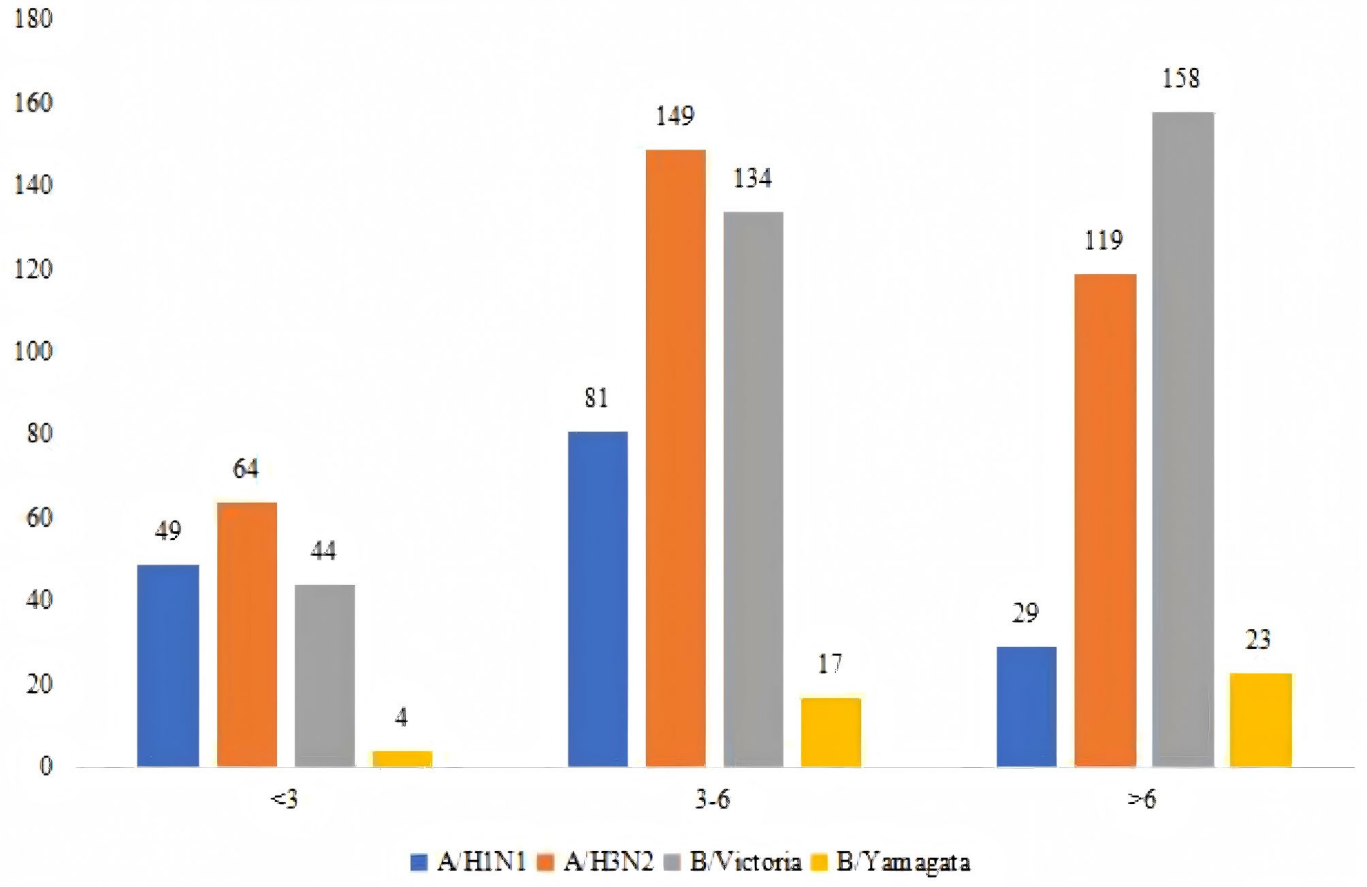
Virus types in different age groups

The influenza-positive rates were higher in patients aged more than 6 years (23.23%) than in those less than 3 years (7.54%) and 3–6 years (13.18%), indicating that the positive rate increased with age (*P* < 0.001) (Table 2). The distribution of the subtypes of influenza virus in the different age groups showed that A/H3N2, A/H1N1, and B/Victoria were common subtypes in patients less than 6 years old; while A/H3N2 and B/Victoria were common in patients more than 6 years; as for B/Yamagata, it only took a small proportion of all the infections. A/H3N2 showed the highest positive rate in the less than 3 and 3–6 age groups, and B/Victoria showed the highest positive rate in the more than 6 years age group (Fig. 2).

**Table 2:** The positive rates of influenza virus in different age groups in 2017–2022

Age group	Influenza-like symptoms, *n*	Positive cases, *n*	Positive rate*, %	*P*
< 3 years	2335	176	7.54	< 0.001
3–6 years	3011	397	13.18	
> 6 years	1429	332	23.23	
Total	6775	905	13.36	

*: The positive rate was calculated as the number of positive cases divided by the number of influenza-like symptom cases

**Fig. 3:**
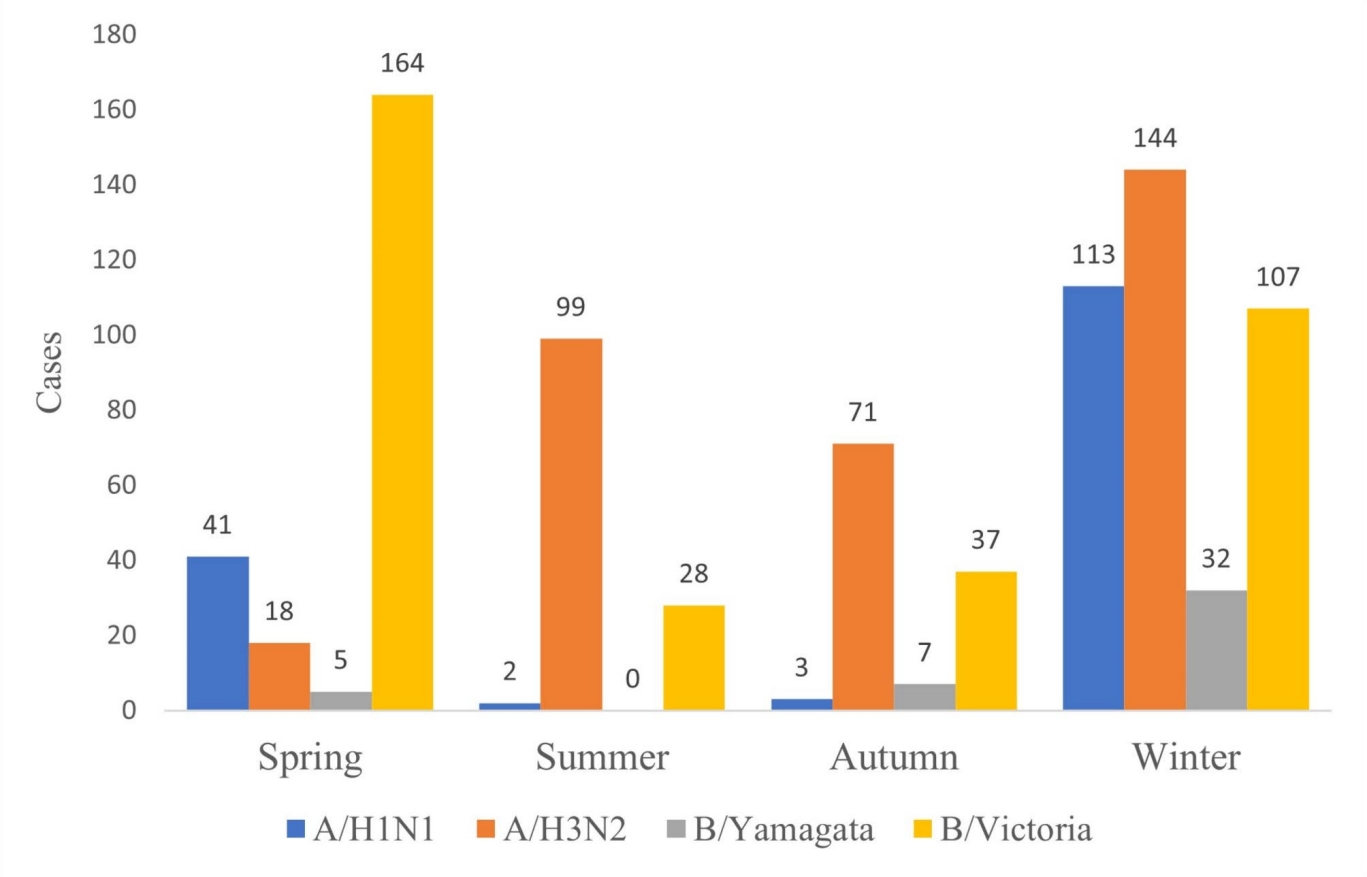
Influenza types and different seasons in 2017–2022

**Fig. 4:**
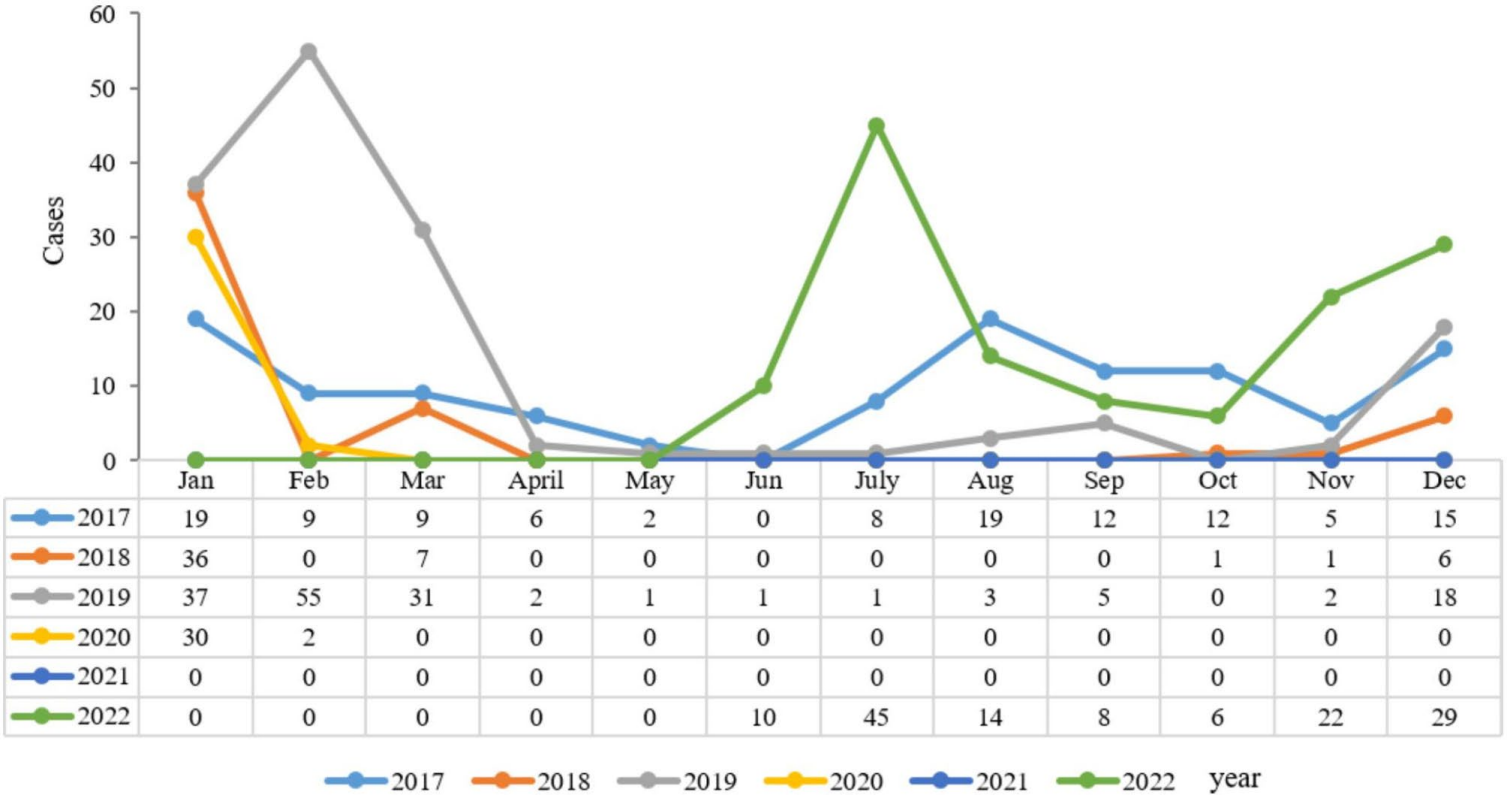
Influenza A epidemiological trends in 2017–2022

**Fig. 5:**
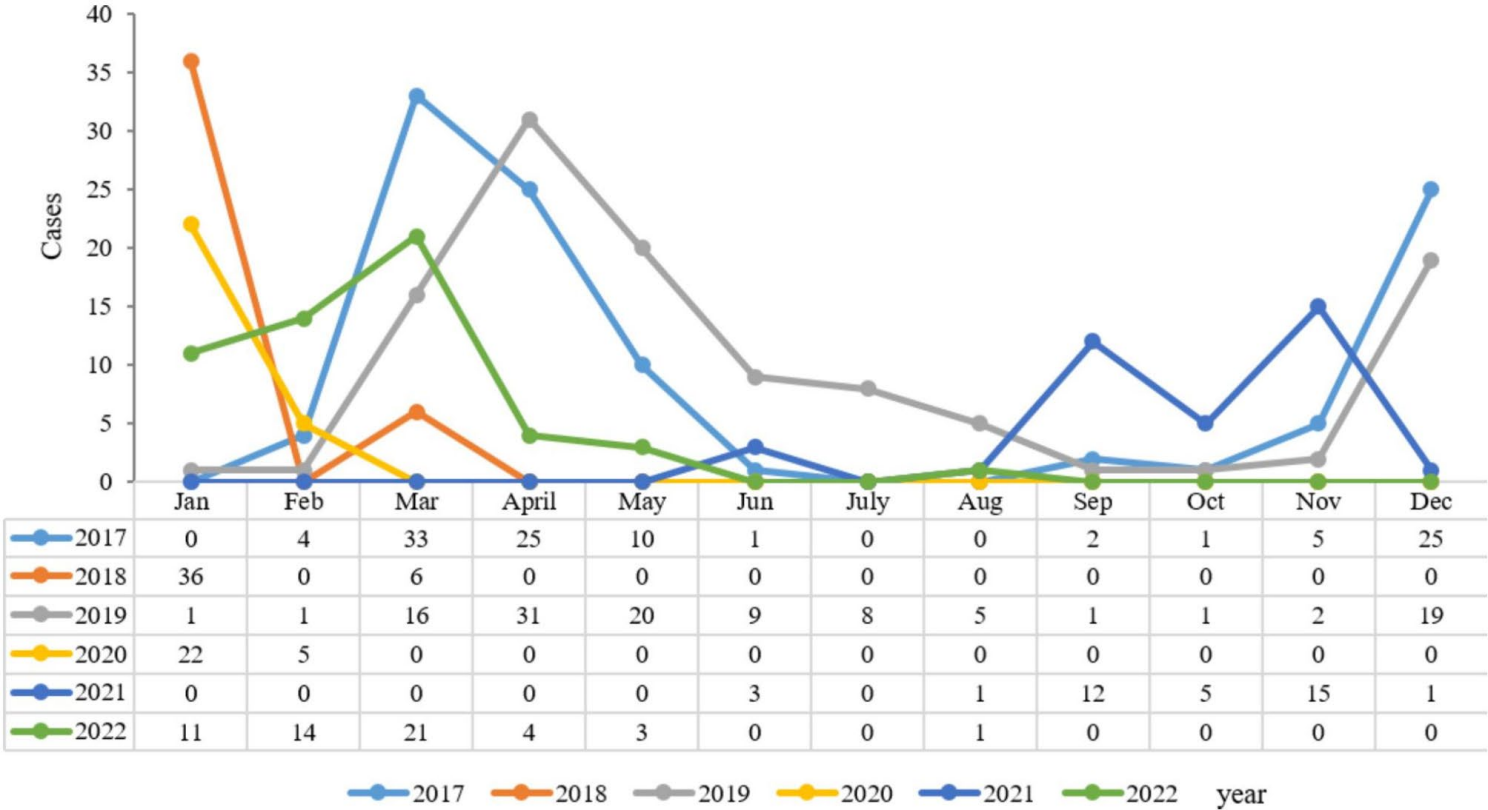
Influenza B epidemiological trends in 2017–2022

The positive rates of influenza A (7.41% vs. 7.22%, *P* = 0.767) and B (5.47% vs. 5.94%, *P* = 0.407) among boys and girls did not have much difference (Table 3). The seasonal characteristics of the influenza infections showed that, among patients with influenza-like symptoms, winter (*n* = 431, 27.88%) and spring (*n* = 227, 12.99%) were the dominant flu season in Hangzhou, and a significant difference of positive rate among seasons were observed (*P* < 0.001). The influenza positive rates among different seasons were different, the positive rates in winter were higher compared with the other seasons except for 2021 and 2022 (Table 4), more specific, in Winter, all the four viruses showed high prevalence, with A/H1N1, A/H3N2, and B/Yamagata reached the top, while in spring the B/Victoria was the most common subtype, this study also revealed that A/H3N2 was also predominant in summer (5.74%) (Fig. 3 and Supplementary Table 1). The epidemiological trends of influenza A and B between 2017 and 2022 are shown in Figs. 4 and 5, respectively.

**Table 3:** Influenza virus positive rate in boys and girls group

	Total, *n* (%)	Positive cases and positive rate, *n* (%)	Negative cases and negative rate, *n* (%)	*P*
Influenza type A				0.767
Boy	3618 (54.16)	268 (7.41)	3350 (92.59)	
Girl	3062 (45.84)	221 (7.22)	2841 (92.78)	
Influenza type B				0.407
Boy	3618 (54.16)	198 (5.47)	3420 (94.53)	
Girl	3062 (45.84)	182 (5.94)	2880 (94.06)	

**Table 4:** The positive rates of influenza virus in different season in 2017–2022

Season	Influenza-like symptoms, *n*	Positive cases, *n*	Positive rate*, %	*P*
Spring	1,747	227	12.99	< 0.001
Summer	1,726	129	7.47	
Autumn	1,756	118	6.72	
Winter	1,546	431	27.88	
Total	6775	905	13.36	

*: The positive rate was calculated as the number of positive cases divided by the number of influenza-like symptom cases

## Discussion

This study showed that influenza viruses infection rate varies during the past 6 years, ranging from 3.03 to 25.7%, and an increased positive rate with age was observed. The most common virus subtypes were B/Victoria in the spring and A/H3N2 in other seasons. Apart from the high prevalence during winter, there was also a summer peak of influenza caused by A/H3N2, suggesting that more attention should be paid to influenza infections during both summer and winter. These findings might provide data support for preventing and controlling childhood influenza and regarding the evolution of influenza strains in children in China.

Influenza causes various illnesses in children, from uncomplicated self-limited illness to severe disease and even death [3–5, 25]. The present study provides an update on the burden of influenza on Chinese children over recent years in Hangzhou City. The overall detection rate of the influenza virus in children with influenza-like symptoms between 2017 and 2022 was 13.36%, slightly lower than the 14.1% observed in 2010–2015 [26]. At the same hospital, from 2016 May to April 2017, Ye et al. [27] reported the detection of the influenza virus antigen using the colloidal gold method from the pharyngeal swab specimens of 34,961 outpatients with fever; the positive rates of influenza A and B were 8.0% and 9.9%, respectively. In 2018, Li et al. [28] reported that among hospitalized children with viral respiratory tract infections, the positive rates for influenza A and B were 19.5% and 4.7%, respectively. These results show the high burden of influenza in the region in recent years.

The present study revealed that the proportion of children testing positive for influenza increased with age, suggesting that children at 3 years old or older (preschool and school-age) were more likely to test positive for influenza, and this phenomenon could be partially explained by the increased exposure to the external environment in > 3 years old children, school is a perfect scenario for influenza circulating and disseminating, prolonged stay at study is associated with the increased prevalence of influenza or influenza-like illness [29]. Considering the effectiveness of children’s vaccination [30, 31], high-risk children above 3 years old (pre-school and school-age) were recommended to receive vaccination before the influenza season. Influenza vaccination effectively reduces the incidence and severity of respiratory infection in children [32]. In the present 6-year influenza surveillance, the most prevalent viruses in 2017 were A/H3N2 and B/Victoria, while in 2018, it was A/H1N1, and in 2019 they included A/H3N2, A/H1N1, and B/Victoria. During 2020–2022, the most prevalent viruses were A/H3N2 and B/Victoria. As for influenza B/Yamagata, in 2017 and 2018, about 20 cases were observed every year, however, from 2019 to 2022, only one influenza B/Yamagata case was observed. In children younger than 3 years and those aged 3–6, the major influenza viruses were A/H3N2, while the major influenza virus was B/Victoria in children > 6 years old. Influenza B/Yamagata infection was observed in all age groups, suggesting using a quadrivalent vaccine, including influenza B/Yamagata. Early antiviral treatment is an important adjunct to influenza vaccination and is recommended for children with suspected or confirmed influenza. There have been many outbreaks in primary and secondary schools and childcare centers in 2017–2019, but fewer in 2020–2022 due to the COVID-19 sanitary measures. Therefore, preventive anti-flu treatment can be given to these children, and if necessary, school and childcare center temporary closure can be considered to reduce contamination. It could also be recommended that public health agencies in the region work to increase health literacy and increase prevention awareness.

Many studies have shown that the prevalence of influenza exhibited a seasonal change pattern, Li et al. reported that influenza peaked during late winter and early spring [33]. In the present study, continuous 60-month surveillance and analysis of influenza showed that winter had the highest overall incidence of documented influenza, but in August 2017, there was a peak of influenza A during summer, and the influenza virus A positive rate reached 21.1%, this atypical peak was in alignment with some previous studies, Sunagawa et al. reported in Okinawa that several outbreaks of A/H3N2 were observed in summer during 2007–2014 [34]; Tsou et al. reported that in 2016/2017, Taiwan has been characterised by an unusual summer peak of A/H3N2 [35]. This atypical peak underlines the importance of monitoring influenza outbreaks in real-time and adjusting the public health response accordingly. Influenza B may be prevalent with influenza A or slightly later than influenza A, and the prevalence of influenza B can be even higher than influenza A and last longer. There are minor differences in the distribution of signs and symptoms across influenza virus types and subtypes/lineages [21], but oseltamivir does not appear to be as effective against influenza B as against influenza A in clinical practice, highlighting the need for surveillance of the influenza types [36]. There were no differences among seasons in 2020–2021, probably due to COVID-19 sanitary protocols.

Mosnier et al. [21] reported that boys were less likely to be infected with influenza B viruses. The present study showed that the positive rate of influenza B was 8.0% in boys and 10.0% in girls (χ^2^ = 3.82, *p* = 0.051). Therefore, more clinical data are needed before a conclusion can be drawn.

This study had limitations. For convenience, only about 20 cases were selected each week for surveillance during the study period, therefore no clinical symptoms and vaccine information were not available for further analysis. Although the prevalence patterns of influenza during that period can be estimated, the exact real prevalence cannot be determined. Also, the treatment outcomes and mortality could not be analyzed using the available data. The surveillance was performed in only one city in China.

## Conclusions

The influenza viruses infection rate in 3 months to 14 years participants ranged from 3.03 to 25.7%, and the positive rate increased with age. No significant difference was observed in different sexes and subtypes of influenza. However, the relatively high prevalence of influenza A/H3N2 and high prevalence of all four subtypes indicates more attention to influenza infection should be paid in summer and winter.

## Electronic supplementary material

Below is the link to the electronic supplementary material.

Supplementary Material 1

## Data Availability

All data generated or analysed during this study are included in this published article.
