# Supplementary material for: Epidemiology of influenza from 2017 to 2022 in a national children’s regional medical center
Source: BMC Pediatr. 2025 Mar 27;25:240. doi: 10.1186/s12887-025-05416-y (PMC11948843; doi:10.1186/s12887-025-05416-y)
Supplement: Supplementary file 1 — Supplementary Material 1 [file 12887_2025_5416_MOESM1_ESM.docx]

Supplementary Table 1. The positive rates of different influenza virus in different season

| **Season** | **Influenza virus** | **Positive cases, n** | **Positive rate*, %** |
| --- | --- | --- | --- |
| Spring (n=1,747) |  |  |  |
|  | A/H1N1 | 41 | 2.36 |
|  | A/H3N2 | 18 | 1.04 |
|  | B/Yamagata | 5 | 0.29 |
|  | B/Victoria | 164 | 9.44 |
| Summer (n=1726) |  |  |  |
|  | A/H1N1 | 2 | 0.12 |
|  | A/H3N2 | 99 | 5.74 |
|  | B/Yamagata | 0 | 0 |
|  | B/Victoria | 28 | 1.62 |
| Autumn (1756) |  |  |  |
|  | A/H1N1 | 3 | 0.17 |
|  | A/H3N2 | 71 | 4.04 |
|  | B/Yamagata | 7 | 0.40 |
|  | B/Victoria | 37 | 2.11 |
| Winter (1546) |  |  |  |
|  | A/H1N1 | 113 | 7.73 |
|  | A/H3N2 | 144 | 9.86 |
|  | B/Yamagata | 32 | 2.19 |
|  | B/Victoria | 107 | 7.32 |

*: The positive rate was calculated as the number of positive cases divided by the number of influenza-like symptom cases.
